# Body Composition, Nutritional Intake Assessment, and Perceptions about Diet for Health and Performance: An Exploratory Study for Senior Futsal Players

**DOI:** 10.3390/nu15061428

**Published:** 2023-03-16

**Authors:** Sílvia Zambujo Brum, Bela Franchini, Ana Pinto Moura

**Affiliations:** 1 DCeT, Universidade Aberta, 4200-055 Porto, Portugal; 2 Faculty of Nutrition and Food Sciences, University of Porto, 4150-180 Porto, Portugal; 3 GreenUPorto—Sustainable Agrifood Production Research Centre/Inov4Agro, 4485-646 Vila do Conde, Portugal

**Keywords:** anthropometric data, athlete, diet, qualitative research, self-reported dietary intake, sport

## Abstract

This study aims to assess the body composition and nutritional intake of senior male futsal players from the II Futsal Division—Azores Series and explore their individual viewpoints regarding the benefits and barriers of healthy eating and performance. Two groups were identified: those who only completed the sociodemographic questionnaire and the anthropometric data (Group 1, n = 48), and those who additionally had their food intake assessed using three 24-h dietary recalls and were interviewed (Group 2, n = 20). Although most of the players have a healthy body composition, those from Group 2 had a significantly higher Body Mass Index, showing that they are under “pre-obesity”, and have a higher percentage of body fat compared to the players from Group 1. Findings from the nutritional intake assessment revealed that players from Group 2 met dietary recommendations for protein, but not for energy and carbohydrate, and they slightly exceeded recommendations for fat. Findings from the interviews revealed that most of these players reported low levels of satisfaction with their sport performance, explained by their deviation from a healthy eating practice in their daily lives. They recognized the need to alter their diets, identifying food items that should be taken and avoided.

## 1. Introduction

Futsal, or indoor soccer, is a team sport characterized by its high intensity, with intermittent and acyclic activity. It demands strength levels for shots, starts, quick changes of direction and the ability to repeatedly sprint during the game, which simultaneously requires the aerobic and anaerobic energy systems [1,2,3,4,5]. These characteristics require a high physical, technical and tactical capability. The body composition [6,7], the power of the lower limbs, the oxygen uptake, the range of motion, the repeated sprint ability, anaerobic fitness and passing skill, agility and coordination can contribute to the optimum performance of the futsal player [1,2]. These performance factors can be affected by the athlete’s body composition, as it is well-accepted that, for example, excessive body fat can potentially impair performance in team sports and, conversely, a greater percentage of muscle skeletal mass tends to increase sport performance as it contributes to energy production during high-intensity activities and enhances players’ force production capabilities [8]. According to the systematic review by Spyrou et al. [3], futsal players display a low percentage of fat, between professional and semi-professional players.

Therefore, these physiological demands involve specific nutritional requirements of each athlete according to their age, sex, team sport and position. Several organizations have been establishing guidelines for nutritional requirements for team sports to better improve sports performance [9], even though nutritional guidelines for futsal were not identified in the literature. Deficiencies in energy can have implications for players’ performance, including a loss of muscle mass, disturbances to immune function, decreased bone mineral density, increased susceptibility to injury and increased prevalence of symptoms of overtraining [10]. Moreover, players who do not achieve energy recommendations or an appropriate balance of macronutrients may find that this does not allow for training adaptations and recovery. However, according to systematic reviews of energy and macronutrient intakes of team sport athletes, athletes tend not to comply with general dietary recommendations, particularly for fat, carbohydrates, and energy intake [9,11]. Thus, it is important to better understand the main barriers that hamper players’ eating habits for better health and performance [12,13,14].

This research aims to assess the anthropometric data and the nutritional intake of senior male futsal players of the II Futsal Division—Azores Series. These results may be useful to determine what could be changed to improve player performance. It also generates data for comparison in future research in the field of athletes’ body composition, nutritional intake, and performance. Additionally, this research aims to explore the players’ perceptions of benefits and barriers to healthy eating, and their influence on performance. These factors should be considered when developing strategies to support senior male futsal players to eat optimally for health and performance at home, work and in sports environments.

## 2. Materials and Methods

### 2.1. Participants and Study Design

Participants were senior male futsal players of the National Championship Futsal Division II—Azores Series. In August 2018 (season 2018/2019), this consisted of eight teams, of which only five were assessed for this research. The teams included 161 players of the Football Association of Angra do Heroísmo, registered in the Portuguese Football Federation [15]. From 161 players, only 68 agreed to participate in this study. The League runs a season from August until May, with a pre-season tournament that starts in the second week of September until the third or fourth week of October, and the league starts usually in the first week of November, going until mid-May.

To achieve the proposed research aims, the research was comprised of two different phases that were carried out between August and November 2018. The first phase consisted of the assessment of the futsal players’ body composition and sociodemographic data. In the second phase, futsal players’ nutritional intake was measured, and they were interviewed face-to-face to better understand their eating habits and their previously obtained anthropometric data.

The study began with different sessions, one for each team, in which the research aims were clearly explained to the futsal players. In these open/clarification sessions, participants were also invited to further participate in the anthropometric, 24-h dietary recall and interview sessions. They were instructed on how they should correctly complete the self-administered 24-h dietary recall. At the end of these sessions, the printed 24-h dietary recall questionnaires were distributed to the players, and they also filled out a brief sociodemographic questionnaire. Following the Helsinki statement, the study was approved by the Football Association of Angra do Heroísmo, as well as by each of the five different clubs in which the study was carried out, for ethical clearance. Informed consent was obtained from all the futsal players and all data were processed under rigorous anonymity, guaranteeing adequate Data Protection procedures.

### 2.2. Instruments and Procedures

#### 2.2.1. Phase 1: Futsal Players’ Body Composition and Sociodemographic Data

Anthropometric data were collected by the first author, a qualified nutritionist, and were collected following the protocol of The International Society for the Advancement of Kinanthropometry (ISAK) [16]. Following this, height was measured using a Seca^®^ stadiometer (cm, sensitivity 0.1 cm), weight using a Tanita^®^ BC-606 scale (kg, sensitivity 0.1 kg) and thickness of three skinfolds (tricipital, abdominal and thigh) in mm with a Jamar Medical Sammons Preston^®^ skinfold calliper (sensitivity 1 mm). For each athlete, triple anthropometric measurements were obtained and the mean of the three measures was used to calculate the sum of each of the three skinfolds.

The anthropometric measurements were carried out in a reserved room in the training halls for greater privacy of the players. The measurements were taken before their training sessions, at the same time, from 7 p.m. to 9 p.m.

The brief sociodemographic questionnaire considered the following variables: age, education, and occupation.

#### 2.2.2. Phase 2: Futsal Players’ Nutrition Intake Assessment and Perception of Diet and Its Role on Sport Performance

The futsal players’ dietary assessments relied on a 24-h dietary recall, for which players were invited to auto-record all food and drinks that they consumed for three different days, corresponding to “training”, “rest” and “competition” periods. This was undertaken because the dietary needs of players vary from day to day, depending on the activity [17]. The 24-h dietary recall applied was adapted from the National Food and Physical Activity Survey (IAN-AF) and contained guidelines for correct completion, prompting the athlete to mark down everything he had ingested, including sips or tests [18]. The questionnaire contained three tables to be filled in on the different days requested (rest, training, and competition), with the following data: date, time of waking up and going to bed, mealtime, meal, place, who they had the meal with, quantity, unit, food/drink, brand and method of cooking. It also contained images based on homemade measures for food quantification, adapted from the IAN-AF Food Quantification Manual, and a possible intake for one day was provided as an example. The athlete was asked not to change their food consumption because they were recording their intake [18].

Face-to-face semi-structured interviews were the method of choice to obtain in-depth descriptions of the diet that players practice (regarding healthy eating habits) and their opinions about how they relate the impact of eating to their sports performance. A semi-structured interview guide of open-ended questions was developed, considering the following dimensions: (i). perceptions of healthy eating, (ii). benefits and barriers towards eating healthily and (iii). the role of food and diet on health and sports performance.

The players were interviewed individually by the first author, at a time and place of their choice, usually in sports halls at the end of the day, before the participants’ training. All interviews were audio-recorded. They were anonymously transcribed verbatim and then handed to the participants to be read and validated.

### 2.3. Data Analysis

The percentage of fat mass (FM) was calculated according to the improved equation developed by Giro et al. [19] for futsal players and the resulting subject health status was classified according to Gallagher et al.’s method [20]. The Body Mass Index (BMI) was calculated using the Quetelet index: body mass/height^2^ (kg/m^2^), according to the WHO classification [21]. The Mann–Whitney U test was used for comparison between player groups, regarding their participation along the different phases of the research.

Energy and macronutrient intakes were estimated using the Nutrium^®^ software v.2019 converting food into nutrients, using homemade measures and, in the case of whole packaged food and beverages, the corresponding amount. Additionally, recipes were also considered. The average intake of energy and macronutrients was calculated by assessing the different 24-h recall data, considering that athletes followed a regular weekly schedule with three days of training, three days of rest and one day of competition [20]. Results regarding anthropometric, body composition and nutritional intake data are expressed as mean ± standard deviation (SD), maximum and minimum. The level of significance was set at *p* < 0.05. The IBM SPSS Statistics v. 25.0 software package was used to analyse data.

Interviews were analysed using a thematic analysis procedure [22]. Themes were identified inductively, and the content was analysed both in terms of manifest and latent themes, an analytical process that involves a progression from description to interpretation of data [23]. The transcripts of the interviews were processed through a qualitative data analysis software, QSR NVivo 12Pro^®^, and a comprehensive process of data coding and identification of themes, consistencies and discrepancies across themes was undertaken and explored to provide an in-depth understanding of the texts. Extracts were not exclusively assigned to separate themes and the overlap between themes in the data was used to inform the broader analysis [22]. Once groups of themes were created, constant comparison was used on the internal homogeneity and external heterogeneity of the categories [24]. To support the analysis, calculation of the number of participants who mentioned a particular theme was performed as the best indicator of a prevalent theme [25]. To illustrate the analysis, direct quotes by the participants were transcribed, serving as a description of the topic explored. The quotes used in this text were translated into English.

## 3. Results

### 3.1. Sample Characterization

A total of 68 futsal players’ body composition and sociodemographic data were collected. All participants were male, the majority were young adults, aged between 17 and 39 years. The sample is mostly composed of single individuals, with a higher percentage of participants having a low education level education and being professionally occupied (Table 1).

Two groups of futsal players were identified, considering their participation along the different phases of the research: (i). those who only completed the sociodemographic and the anthropometric data (n = 48, Group 1—partial participation) and (ii). those who additionally had their food intake assessed using the three 24-h dietary recalls and were later interviewed (n = 20, Group 2—full participation).

### 3.2. The Players’ Body Composition

Altogether, our players have a “normal weight”, as they presented a mean of 24.7 ± 3.5 kg/m^2^ of BMI and have their FM in a healthy state (Table 2). However, comparison between the two groups confirmed that players from Group 2 have significantly higher BMI (26.8 ± 4.8 kg/m^2^), showing that they are under the “pre-obesity” nutrition status. Although both groups have a healthy FM, futsal players belonging to Group 2 also have a higher FM (15.6%) compared to the players from Group 1 (Table 2).

### 3.3. Nutritional Intake Assessment

Table 3 considers the average intake of energy and macronutrients from the three days from the 20 futsal players who responded to the three 24-h dietary recall questionnaires: three days of training, three days of rest and one day of competition. Overall, players reported consuming a mean total daily energy intake of 2445 kcal, which was a slight underconsumption regarding the ISSN energy requirements for athletes [26]. The carbohydrate intake (3.4 g/kg of body weight), which contributed 41.7% of total energy (TE), fell below the ISSN recommended intakes of 5 to 8 g/kg/day [26].

The average protein intake was 1.9 g/kg of body weight, within the recommendations for athletes according to Jäger et al. [28], which can vary between 1.4 g/kg at 2.0 g/kg of body weight. The average lipids intake was 83.9 g, contributing to 30.1% of total energy (TE), slightly exceeding the recommended fat intake [26].

### 3.4. Interviews: Futsal Perception of Diet and Its Role in Futsal Practice

Two broad levels of analysis were identified, which combined many dimensions, cutting across the different topics of discussion: (i). eating habits and (ii). sport performance. All 20 participants from Group 2 discussed these two broad levels in their interviews. The eating habits topic represented the internal forces that influence the players’ eating behaviours. The sports performance level considered the possible relations that our players perceived between diet and futsal practice.

#### 3.4.1. Healthy Eating: Perceived Concept, Benefits and Barriers

When asking our players to explain what “healthy eating” meant to them, they listed specific food items that should be eaten (e.g., F and V, fish and meat and to a lesser extent, water and dairy products), and those that should be avoided or taken in lesser quantities, namely high energy density foods such as fried foods, fast food, burgers, sugar and foods with added sugar; and to a lesser extent alcoholic drinks, cereals and derivatives and salt. To achieve healthy eating, some of our players also referred to the idea of a balanced and varied diet, allowing them to obtain different nutrients, such as protein, vitamins or carbohydrates “…that are necessary for an all-day” (P90). To a small extent (n = 3), healthy eating was discussed in terms of cooking techniques, where it was found that grilled foods were considered healthier than fried foods.

For our players, food is a crucial aspect of their lives, and 75% of them believed that compared to those who do not practice sports: “… the players should have attentive care of their diet”, P79, because “practicing a sport incites other necessities” P63. Eating healthy allows them to have better wellbeing (“The greatest benefit is the physical well-being that we feel”, P65), and enables them to have better sport performance (“Because an athlete needs to have strength to run or whatever else the sport demands, the athlete might need more nutrients and more…”, P46) and weight control: “There are two benefits that are essential to me. Firstly, weight control: in my practice I feel that weight control boosts my output not only in my athletic endeavours but in my day-to-day life. The other benefit I see is health”, P01.

Despite these perceived, strongly linked benefits, only 35% of the 20 players interviewed believed that they eat healthily, 45% of them mentioned that they deviate from healthy eating practices in their daily lives, and 20% of them reported that their healthy eating practices are not always consistent, as they eat healthily “sometimes”, P52, or as said P63: “more or less, … I’ve may days”. Additionally, most of our interviewed players (n = 19/20) reported that sometimes they eat too much, particularly on the weekends, summer holidays or other special occasions. In this context, our participants identified different related barriers that hamper healthy eating practices (Table 4). Nevertheless, two main barriers emerged among the opinions of our players: lack of availability of healthy foods and lack of time to prepare healthy meals. In fact, as most of our participants were young adults and actively working (Table 1), some of them experienced difficulties: “… to find healthy food to eat at working place”, P59. Others, especially younger players, recognized that their parents or other family members (e.g., sister or grandmother) are often responsible for the food preparation in their households, limiting their influence by not allowing them to participate much in labour-intensive meals: “…my mother makes the food I eat whatever is made…” P05. Others, on the other hand, perceived time pressure as a main barrier, and may think that they do not have time to prepare healthy meals when eating at home, instead seeking out convenience food, as reported by P65: “…many times I have to make some quick food, or I need something convenient to eat”. Unhealthy habits, difficulties in giving up favourite foods (e.g., chocolates, cakes) and economic constraints were also barriers cited by our players (Table 4).

#### 3.4.2. Sport Performance: Perceived Body Shape and Strategies to Improve Performance

In general, our players were not used to assessing their own body composition, as only 35% of them (n = 7/20) reported performing assessments. The main parameters assessed by these players, performed at different time intervals (daily, weekly, or monthly), were weight, lean mass, FM, BMI and how their clothes fit. They explained that these measurements allowed them to control the development of their body parameters to better adapt eating and training: “To see if everything is within what I consider to be a standard for an athlete… to evaluate above all a continuity of what I am accomplishing in the gym or with my diet”, P79. The other players (n = 13/20) reported that they were not used to self-assessing their own body composition, essentially for lack of interest (n = 10, “I never gained the habit…”, P46; “Sometimes I just don’t remember…”, P65; “I’m not really comfortable with my body right now…”, P84), and to a lesser extent, for inadequate conditions or instruments to do it (n = 2, “… I don’t have a place to do it…”, P65, “…I don’t have a scale at home…” P18). Additionally, 15 of them recognized low levels of satisfaction with their sport performance, compared to four players who expressed that they are in good shape. As a result, they essentially expressed worries about food and performance associations, in terms of fatigue, weight gain and general malaise (Table 5).

Thus, to cope with this dissonance, our players identified different strategies that may improve their sports performance, namely: improving eating habits, training more, losing weight, motivation, resting more and supplementation (Table 6). Typically, the dominant strategy for improving sports performance focuses on how to improve eating habits. For this, they identified specific foods that influence their performance in a negative way, and thus should be avoided (e.g., red meat protein, sugar and foods with added sugar, foods with high energy density, dairy products, alcoholic beverages, and salt). Food items that influence their performance in a positive way, and thus should be eaten, were also identified: meat and fish, cereals and derivatives, F and V and dairy products. Curiously, supplementation was not a hot topic for our players, as only nine out of the twenty participants reported having used supplementation and only one of them considered supplementation as a possibility for improving sports performance (Table 6).

## 4. Discussion

As for basic anthropometric measurements, the senior futsal players showed a mean height of 1.74 ± 0.07 m and a mean weight of 75.2 ± 12 kg. These results were similar to the means found in other studies with Portuguese futsal players: 1.75 ± 0.07 m and 71.5 ± 7.6 kg [29], and 1.76 ± 0.08 m and 72.8 ± 1.0 kg [19].

This study also shows that players are globally eutrophic (BMI 24.7 ± 3.5 kg/m^2^) and have an average FM of 19.1 ± 4.0%, which may be considered slightly higher, but within range, when compared with other professional and semi-professional Portuguese futsal players, who present an average FM of 15.8 ± 3.2% [19]. In fact, in futsal practice, lower FM tends to favour maximum player performance, as this sport demands high-intensity and intermittent actions that require high physical effort from the players [1]. The excess FM causes greater energy expenditure, therefore hindering the post-exertion recovery process [30]. When compared with the literature, the players in this study showed higher triceps skinfold (13.3 ± 5.5) compared to Médici et al. and Stubbs-Gutierrez et al. [31,32] The thigh skinfold (15.9 ± 6.0) was similar to Médici et al. and Giro et al. [19,31] and different from Stubbs-Gutierrez et al. and Bonatto et al. [32,33]. The abdominal skinfold (17.7 ± 7.0 mm) was similar to Médici et al. and Bonatto et al. [31,33] and higher compared to Giro et al. and Stubbs-Gutierrez et al. [19,32].

After dividing the sample by those who fulfilled all the research phases, differences were observed between Group 1 and Group 2. The players from Group 2 had significantly higher BMIs (26.8 ± 4.8 kg/m^2^, *p* = 0.003), compared to the players of Group 1, showing that they are under the “pre-obesity” ponderal status. In the same way, significant differences were observed in the abdominal skinfold (*p* = 0.035), which was greater in Group 2 (20.7 ± 8.0 mm) than in Group 1 (16.5 ± 6.7 mm).

Following the results of the majority of studies on sport nutrition [9], our findings show that the total energy and carbohydrate intakes of Group 1 players did not meet sports nutrition recommendations for energy and carbohydrate (i.e., ISSN) [26]. In contrast, they slightly exceeded or met recommendations for fat and protein, respectively (ISSN, ACSM) [26,27]. Considering alcohol consumption, the present study confirms a great dispersion of alcohol intake (ranging from 0–145 g), reinforcing that this is one of the most common dietary mistakes in a sports environment [33]. As a result, negative consequences on sports performance can be found, since the intake of carbohydrates is essential to maintaining and replenishing glycogen stores in futsal players, with the penalty of not being able to maintain the same high physical intensity. As futsal is a sport characterized by intermittent intensity exercises, both aerobic and anaerobic, it is urgent to maintain a high glycaemic level, with the threat of affected motor performance due to failure of the muscular system [9,11].

This is particularly relevant, as findings from the qualitative phase revealed that most of the interviewed players (79% of participants of Group 2) recognized low levels of satisfaction with their sport performance, which could be explained by their deviation from healthy eating practices in their daily lives. Two main barriers emerged among opinions of our players that may explain why healthy eating practices may not be followed: (i). lack of availability of healthy foods and (ii). lack of time to prepare healthy meals [12,13,14]. According to these players, they are exposed to an environment that hampers their choice of healthy foods. For those who actively work (most of the players, Table 1), the majority of the food service sector keeps focusing more on the hedonic features of consumer demand and less on a nutritious consumer-oriented menu, hampering them in finding healthy foods to eat in their workplaces. At home, especially for younger players, they are not able to influence the meals served, since they eat meals that have already been decided upon without their opinion by other household members. Younger players should have greater involvement in food shopping and meal preparation because the contribution to food tasks—namely preparing meals—is associated with healthier eating. Additionally, people with perceived time pressure may think that they do not have the time to prepare healthy meals and may seek out convenience food solutions (such as eating in restaurants, frozen main courses or ready-made meals) rather than cooking from basic ingredients [34].

Facing a lack of fitness, players from Group 2 stated in the interview sessions that they were not used to assessing their body composition. This reported behaviour could be explained by the fact that our participants are senior players who probably do not have the support of sports dietitians for high-performance nutrition, or of medical staff for monitoring and optimizing fitness, body composition and performance outcomes, as usually happens with players that participate in professional team sports [11].

Additionally, players from Group 2 expressed worries about food and performance associations, resulting from their own experience, in terms of fatigue, weight gain and general malaise. Recognizing the strong link between “eating habits” and “sport performance”, they rely on improving eating habits to improve sports performance [12,13,35] and recognized the need to alter their diets. For this, these players polarized food items as those that should be taken and those that should be avoided, in line with current sports nutrition recommendations, namely the inclusion of fruits, vegetables and lean protein foods, and the avoidance of energy-dense nutrient-poor foods [14]. Moreover, some players described healthy eating as the ingestion of a variety of foods in moderation, in accordance with the Portuguese New Food Guide [36]. In fact, eating healthily enabled them to achieve better wellbeing, better sport performance and weight control. This reinforces that in the sports context, wellbeing is related to the physical health dimension [37] and the negative influence of excess weight on performance [13,14].

This research has some of the following limitations. The sample size enrolled in the 24-h dietary recall for three days is small, hampering the assessment of the dietary intake of the adult senior players population of the II Futsal Division—Azores Series. This information should be taken into consideration to assess whether all the players are complying with the current recommendations; as discussed above, most players of different sports do not comply with the energy and carbohydrate recommendations, which leads to a dietary pattern that hinders their sports performance [9]. As a result, there is a need for future research assessing the dietary intake of futsal players. Although the 24-h dietary recall is considered a valid method and very used for assessing the nutritional intake of the players [38], there is a bias regarding the fact that the estimated portion sizes and intakes could be underestimated or overrated, as they are self-reported, and that it relies on memory [39]. Moreover, as the interviews with the players had a qualitative focus, the findings are not generalizable to the entirety of the II Futsal Division—Azores Series futsal cohort. Furthermore, the perspectives of the multiple stakeholders involved (e.g., coaches, sport directors, players’ family members) were not compared in the present study. Future research is required to explore the factors that influence athletes’ dietary intakes.

## 5. Conclusions

Most of the players have a healthy body composition, as they are globally eutrophic and have an average FM, which is considered normal for futsal players. However, most of those who had their nutritional intake assessed had BMI values outside the normal range, had an inadequate distribution of macronutrients, had low energy and carbohydrate intakes and consumed alcohol. This can be explained by the fact that they reported experiencing significant challenges with healthy food access, time and financial constraints, food preferences and pressures to achieve a specific body shape and sport performance. These factors should be considered when developing strategies to support senior male players to eat optimally for health and sport performance.

## Figures and Tables

**Table 1:** The male players’ sociodemographic characteristics (n = 68).

	Participants (%)
**Age (years)**	
Mean (±SD)	25 (±5)
**Education level**	
No higher education	53 (77.9%)
Higher education	15 (22.1%)
**Marital status**	
Single	53 (77.9%)
Married	15 (22.1%)
**Occupation**	
Employed	49 (72.1%)
Unemployed	12 (17.6%)
Student	7 (10.3%)

**Table 2:** Anthropometric and body composition characteristics of male futsal players, as a function of participants’ phases of research completion.

	Total (n = 68)		Group 1: Futsal Players with Partial Participation (n = 48)		Group 2: Futsal Players with Full Participation (n = 20)		* *p*-Value
	Mean (±SD)	Range [Min; Max]	Mean (±SD)	Range [Min; Max]	Mean (±SD)	Range [Min; Max]	
Weight (kg)	75.2 (±12.0)	[56.6; 120.2]	73.3 (±10.7)	[56.6;117.0]	80.8 (±17.1)	[58.4; 120.2]	0.096
Height (m)	1.74 (±0.07)	[1.62; 1.92]	1.75 (±0.07)	[1.62; 1.92]	1.73 (±0.07)	[1.62; 1.86]	0.377
BMI (kg/m^2^)	24.7 (±3.5)	[19.4; 39.5]	23.9 (±3.2)	[19.4; 36.5]	26.8 (±4.8)	[21.2; 39.5]	0.003
Triceps skinfold (mm)	13.3 (±5.5)	[5.0; 30.0]	12.6 (±4.9)	[5.0; 30.0]	14.2 (±6.2)	[6.0; 28.0]	0.410
Abdominal skinfold (mm)	17.7 (±7.0)	[6.0; 34.0]	16.5 (±6.7)	[6.0; 34.0]	20.7 (±8.0)	[7.0; 34.0]	0.035
Thigh skinfold (mm)	15.9 (±6.4)	[5.0; 34.0]	15.1 (±6.2)	[6.0; 34.0]	16.9 (±6.5)	[5.0; 33.0]	0.266
FM (%)	19.1 (±4.0)	[12.9; 31.0]	18.6 (±3.8)	[12.9; 31.0]	20.3 (±4.2)	[13.6; 30.1]	0.079

* *p*-value based on non-parametric Mann–Whitney test and a value of *p* < 0.05 was considered significant.

**Table 3:** Nutritional intake of the players (daily mean from three training days, three resting days and day of the competition event) (n = 20) in terms of compliance with recommendations.

	Mean (±SD)	Min	Max	%Total Energy (TE) Contribution	Standard	Reference
Energy (kcal)	2445 (±739)	1278	4438	-	50–80 kcal/kg/day	ISSN [26]
Fat (g)	83.9 (±28.1)	45.1	167.1	30.1	20–35% 30%	ACSM [27] ISSN [26]
Carbohydrates (g)	253.3 (±78.1)	158.3	505.0	41.7	45–65%	ISSN [26]
Carbohydrates (g/kg of weight)	3.4 (±1.1)	2.3	6.9	-	5–8 g/kg/day	ISSN [26]
Protein (g)	142.6 (±46.9)	56.1	222.7	23.4	10–35%	ISSN [28]
Protein (g/kg of body weight)	1.9 (±0.6)	0.8	3.0	-	1.4–2.0 g/kg/day	ISSN [28]
Alcohol (g)	13.5 (±33.3)	0.0	145.0	-	-	

ACSM—American College of Sports Medicine; ISSN—International Society for Sport Nutrition.

**Table 4:** Barriers in adopting a healthy diet.

Barrier	N. of Participants	Examples of Sample Quotes
Lack of availability	9	“No, my sister controls everything! I also don’t help preparing the meals. What she makes is what I eat”, P90
Lack of time	8	“… Right now, my life makes it so that it isn’t easy always having a healthy diet… there is always an obstacle from having a daughter… in terms of daily organisation it doesn’t help to make such healthy foods”, P01
Lack of healthy eating habits	4	“… I think that a diet that goes against what might be best for us is deeply rooted within ourselves… Thus, freeing ourselves from these roots is quite complicated…”, P44
Difficulty to give up favourite foods	3	“… I have a big sweet tooth. But… recently I have managed to control the urge and so I am trying to combat it…”, P68
Financial constraints	2	“… prices…the prices of healthier foods are greater…”, P46 “…products that are sold at low prices are many times the worst when it comes to healthy eating”, P26

**Table 5:** Food and performance associations.

Worry	N. of Participants	Examples of Sample Quotes
Fatigue	9	“Since I’m heavier my reaction, for example, to reach the ball will not be as quick as if I had less weight… I’ll tire myself out faster”, P90
Weight gain	7	“Because maybe the foods that I ingest, if they aren’t healthy, might make me gain weight and for example, gaining weight will affect my performance as an athlete”, P46
Malaise	6	“Yes… if I follow a diet… If I make a meal before the game where, and this has happened, the meal has sauces or where the meal is heavier, even if I think “oh the game is only later in the day”, it will have an impact on the game”, P12

**Table 6:** Strategies for improving sports performance.

Strategy	N. of Participants	Examples of Sample Quotes
Improving eating habits	16	“Improve my eating habits… be more consistent…” P68 “Stop eating fries and fried foods…” P49
Training more	7	“…exercising outside of training hours…”, P26
Losing weight	7	“My athletic performance will usually elevate when my diet is well made, and I have an ideal weight… I feel perfectly that when my weight falls below the norm my output is much lower”, P01
Motivation	3	“Having a want to do it… well, if we truly are in sports, we have to want to run well and enjoy doing it…”, P18
Resting more	2	“…better control of my sleep, I know that having a continuous sleep has its benefits”, P26
Supplementation	1	“I could do more training sessions….and dieting…I could use supplementation and train complementary parts of the body that I don’t train during futsal…”, P53

## Data Availability

Data will be available upon request from the corresponding author.
